# Protection by an Oral Disubstituted Hydroxylamine Derivative against Loss of Retinal Ganglion Cell Differentiation following Optic Nerve Crush

**DOI:** 10.1371/journal.pone.0065966

**Published:** 2013-08-05

**Authors:** James D. Lindsey, Karen X. Duong-Polk, Yi Dai, Duy H. Nguyen, Christopher K. Leung, Robert N. Weinreb

**Affiliations:** 1 Hamilton Glaucoma Center and Department of Ophthalmology, University of California San Diego, La Jolla, California, United States of America; 2 Department of Ophthalmology, Fudan University, Shanghai, China; 3 Department of Ophthalmology and Visual Sciences, Chinese University of Hong Kong, Hong Kong, China; Dalhousie University, Canada

## Abstract

Thy-1 is a cell surface protein that is expressed during the differentiation of retinal ganglion cells (RGCs). Optic nerve injury induces progressive loss in the number of RGCs expressing Thy-1. The rate of this loss is fastest during the first week after optic nerve injury and slower in subsequent weeks. This study was undertaken to determine whether oral treatment with a water-soluble N-hydroxy-2,2,6,6-tetramethylpiperidine derivative (OT-440) protects against loss of Thy-1 promoter activation following optic nerve crush and whether this effect targets the earlier quick phase or the later slow phase. The retina of mice expressing cyan fluorescent protein under control of the Thy-1 promoter (Thy1-CFP mice) was imaged using a blue-light confocal scanning laser ophthalmoscope (bCSLO). These mice then received oral OT-440 prepared in cream cheese or dissolved in water, or plain vehicle, for two weeks and were imaged again prior to unilateral optic nerve crush. Treatments and weekly imaging continued for four more weeks. Fluorescent neurons were counted in the same defined retinal areas imaged at each time point in a masked fashion. When the counts at each time point were directly compared, the numbers of fluorescent cells at each time point were greater in the animals that received OT-440 in cream cheese by 8%, 27%, 52% and 60% than in corresponding control animals at 1, 2, 3 and 4 weeks after optic nerve crush. Similar results were obtained when the vehicle was water. Rate analysis indicated the protective effect of OT-440 was greatest during the first two weeks and was maintained in the second two weeks after crush for both the cream cheese vehicle study and water vehicle study. Because most of the fluorescent cells detected by bCSLO are RGCs, these findings suggest that oral OT-440 can either protect against or delay early degenerative responses occurring in RGCs following optic nerve injury.

## Introduction

Loss of retinal ganglion cells (RGCs) is a well-known consequence of optic nerve damage that occurs in a number of diseases including glaucoma, ischemic optic neuropathy and compressive optic neuropathy [1]–[3]. Several days prior to death, injured RGCs cease expression of Thy-1, an RGC marker protein that normally is upregulated during RGC development at the time of synapse formation [4], [5]. Treatments that delay or reduce this loss of Thy-1 expression may facilitate the recovery of compromised RGCs and protect against vision loss.

Direct optic nerve injury or moderately elevated intraocular pressure induce the early formation of reactive oxygen species in RGCs that can be detected within 1 to 6 hours after insult [6], [7]. Moreover, treatment with iron dextran, which enhances oxidative injury, increases the magnitude of RGC loss following optic nerve crush [8]. Conversely, elevating RGC expression of the anti-oxidant enzyme Cu -Zn-superoxide dismutase reduces RGC loss following optic nerve crush [9]. Both the nitroxide Tempol (4-hydroxy-2,2,6,6-tetramethylpiperidin-1-oxyl) and its hydroxylamine form Tempol-H (1,4-dihydroxy-2,2,6,6-tetramethylpiperidine) are superoxide dismutase mimetics [10]. Intraperitoneal (IP) administration of Tempol or more potent Tempol-acyl derivatives can protect against loss of RGCs following partial optic nerve crush [8], [11]. Similarly, IP injection of Tempol-H can protect photoreceptors from acute light damage [12]. Modification of Tempol-H to facilitate oral absorbtion yielded the derivative 4–(4-(1-hydroxy-2,2,6,6-tetramethylpiperidin-4-yloxy)-1,2,5-thiadiazol-3-yl) morpholine hydrochloride (OT-440). It is unknown whether oral administration of OT-440 can reduce or slow the loss of Thy-1 expression in RGCs that occurs following optic nerve injury.

Recently, a modified confocal scanning laser ophthalmoscope (blue-light CSLO or bCSLO) has been developed that allows imaging of fluorescent retinal neurons in mice that express cyan fluorescent protein under the control of the Thy-1 promoter (Thy1-CFP23Jrs mice) [13]. Retrograde tracer studies in these mice showed that most of the brightly fluorescent retinal neurons are RGCs [13]. In addition, these fluorescent retinal cells gradually lose their fluorescence after optic nerve transection or crush [14]. Moreover, the kinetics of fluorescent cell loss following optic nerve crush can be followed in vivo and is similar to the kinetics of Thy-1 protein reduction following optic nerve crush [5], [14]–[16]. This suggests that longitudinal evaluation of the loss of fluorescent retinal neurons in Thy1-CFP23Jrs mice can provide a useful measure of early RGC damage following optic nerve crush.

In view of these considerations, the present study was undertaken to determine whether oral treatment with OT-440 protects against loss of fluorescence in fluorescent retinal neurons of Thy1-CFP23Jrs mice that received optic nerve crush.

## Results

### Control Studies

**Figure 1:**
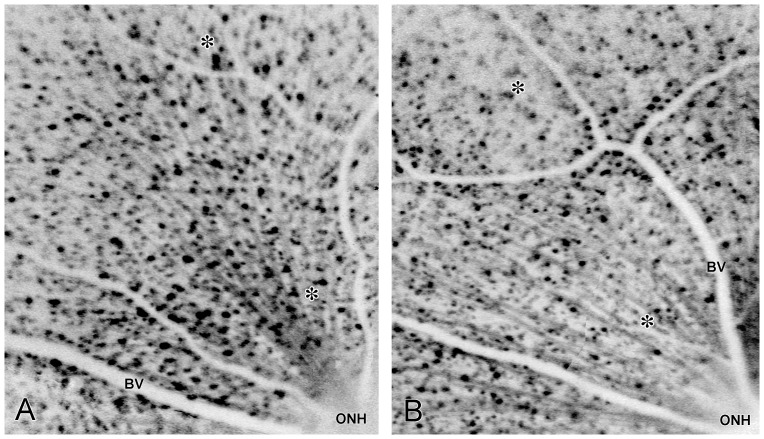
Inverted low magnification montage images of mouse retinas obtained using the blue light scanning laser ophthalmoscope. Fluorescent retinal neurons appear as black spots, nerve fiber layer axon bundles appear as dark streaks radiating away from the optic nerve head (ONH), and blood vessels appear as branched thick white lines indicating their lack of fluorescence (BV). Note the similar organization of these structures in two different eyes (panels A and B). Asterisks in each panel indicate the retinal eccentricity range within which the quantitative analysis regions were obtained.

Retinal bCSLO images were inverted to facilitate identification and counting of fluorescent retinal neurons. These images showed fluorescent retinal neurons as dark spots, nerve fiber layer axon bundles as dark streaks radiating out from the optic nerve head, and branched retinal surface blood vessels as thick white lines indicating their lack of fluorescence (Figure 1). Overall, the organization of these structures were similar among the various study eyes (Figure 1A vs. 1B.) Nevertheless, counts of fluorescent retinal neurons in the same size analysis areas within these baseline images revealed differences between right and left eyes that ranged from 5.3% to 56.6% with a mean difference of 24.0±14.8% (Table 1, mean ± SD, N = 9 eye pairs). Among retinas from different animals, the count variations could be up to 2-fold (compare column entries in Table 1). In view of these results, it seemed appropriate to investigate the differences between the baseline counts of a defined retinal area from an experimental eye and counts of the same retinal area of that eye collected at different time points after optic nerve crush. This approach would be used for each analyzed eye. Hence, we chose to identify analysis regions containing exactly 64 neurons at baseline that were suitable for analysis at the subsequent time points. The bCSLO images for quantitative analysis were similar to those shown in Figures 1A and 1B, and the analysis regions (containing the 64 neurons to start) were selected from the readily visualized regions indicated by the asterisk pairs in Figure 1.

**Table 1:** Variability of baseline counts from contralateral eyes generated using a standardized area.

Animal No.	OD	OS	Percent Difference*
1	62	72	13.9
2	99	43	56.6
3	44	34	22.7
4	74	49	33.8
5	69	85	18.8
6	57	54	5.3
7	51	71	28.2
8	46	54	14.8
9	68	87	21.8
Mean ± SD	63.3±17.0	61.0±18.6	24.0±14.8

* Calculated as 100 – (100× smaller count/larger count) for each pair of eyes.

There were two studies. The first compared OT-440 prepared in cream cheese to cream cheese alone. These animals were allowed to eat each dose from small cups fastened to the inside of their cage and typically completed this task quickly. There were three doses/day and the total dose was 5 mg/day. The second study compared OT-440 dissolved in water to water alone that was delivered by gavage. There were two doses/day and the total dose was 5 mg/day. After the initial baseline images were collected, the treatments were started. The animals were imaged again two weeks later to determine whether there was a direct effect of the treatments on the number of fluorescent retinal neurons. For each of the experimental groups, differences between the baseline counts and the counts of the same retinal areas made after two weeks were insignificant (mean variation <3.0% for each group, *P*>0.35 for each group, paired t test).

All animals then received unilateral optic nerve crush. The vehicle and OT-440 treatments were continued for four more weeks, and the eyes that received optic nerve crush were imaged weekly.

### Qualitative assessment of images collected after optic nerve crush

**Figure 2:**
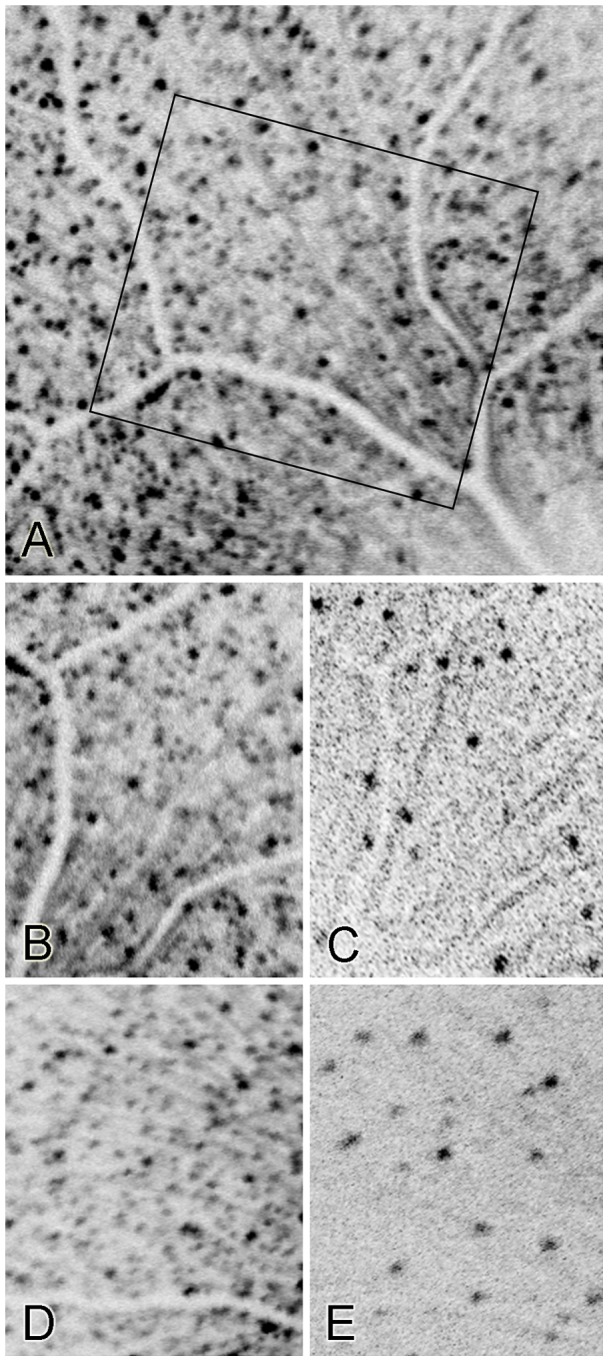
Inverted images of fluorescent retinal neurons imaged at baseline and at 4 weeks after optic nerve crush. Panel A shows a low magnification image to illustrate the origin of the higher magnification images (box). The higher magnification images include image pairs from a mouse that received vehicle cream cheese (B and C) and a mouse that received OT-440 in cream cheese (D and E). Panels B and D are baseline images while panels C and E show the same areas of the same eyes at 4 weeks after optic nerve crush. Note that the remaining fluorescent retinal neurons at 4 weeks can be identified in the baseline images. Also note that these images extend closer to the optic nerve head than the proximal eccentricity limit shown in Figure 1 in order to illustrate that fluorescent retinal neuron loss occurs near the optic nerve head similarly to within the mid-peripheral retina.

In mice that received either vehicle, there was a substantial reduction in the number of fluorescent retinal neurons after optic nerve crush (Figure 2). However, cells still fluorescent at 4 weeks after optic nerve crush were readily identifiable in the corresponding baseline images based on their relative position to blood vessels and other remaining fluorescent retinal neurons. Often the intensity of fluorescence was reduced in the remaining cells. Sometimes, the relative intensity of fluorescence among the remaining fluorescent cells was altered so that certain cells that had been brightly fluorescent become markedly less fluorescent while other cells were less dramatically altered. To maximize the detection of weakly fluorescent cells, the gain of the imager was increased resulting in images with moderately graining background (Figure 2C and 2E). We found this strategy increased the reproducibility of the results [16].

### Quantitative Analysis: OT-440 mixed with cream cheese

**Figure 3:**
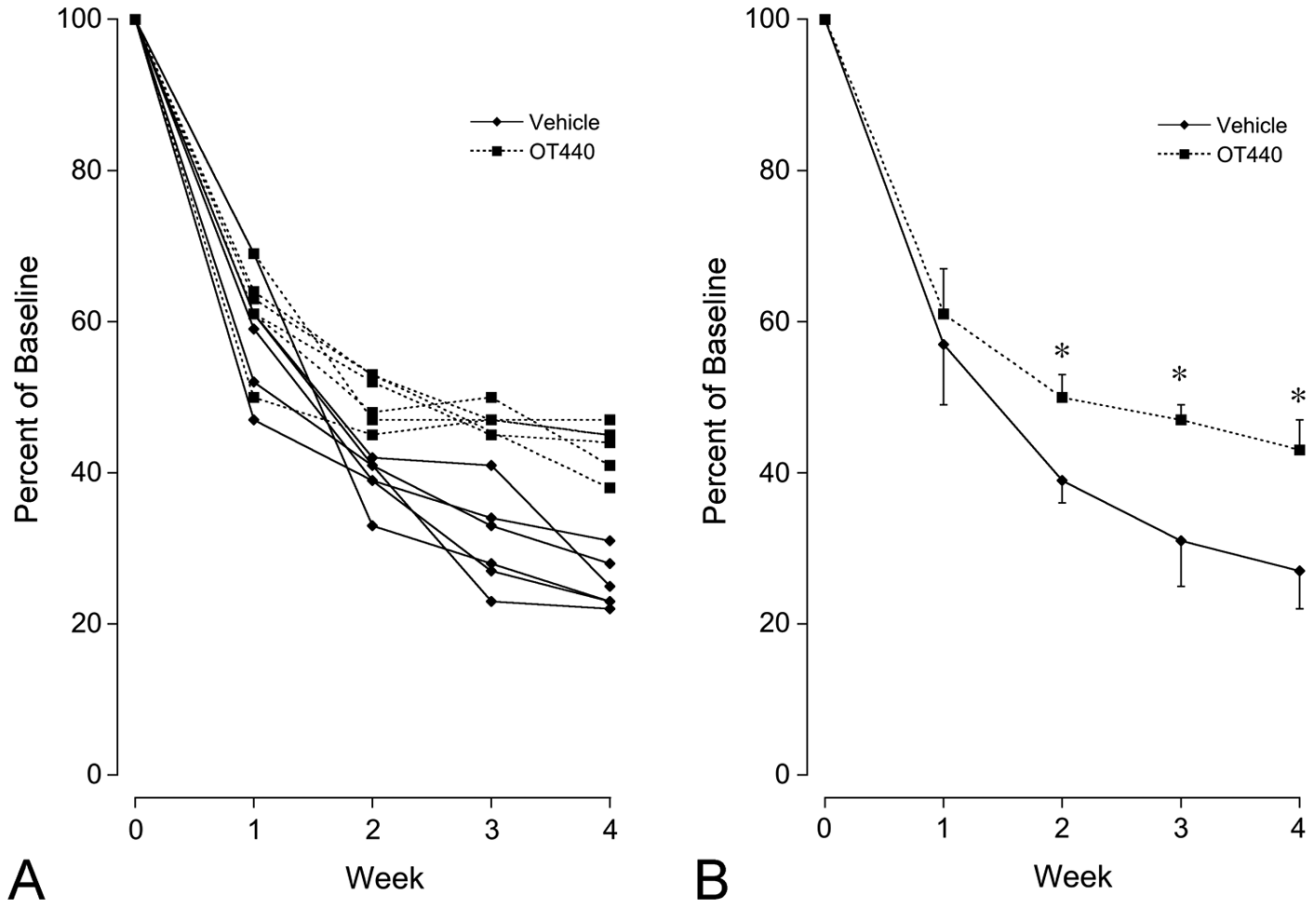
Loss of fluorescent neurons during the four weeks in defined retinal areas following optic nerve crush in mice that received cream cheese vehicle or OT-440 in cream cheese. Total data graph (**A**) shows that the general pattern for each eye was a more rapid decline during the first week of the study followed by slower decline in the subsequent weeks. Mean data graph (**B**) shows significant differences between the vehicle and OT440 groups at weeks 2, 3 and 4. N = 7 for each data point in the vehicle group except for weeks 2 and 3 where N = 6 due to a corneal scratch in one animal that later healed. Similarly, N = 6 for each data point in the OT-440 group except for week 3 where N = 5. Asterisks indicate *P*<0.001 (students t-test). Error bars indicate SD.

Sets of images of defined areas that contained 64 cells at baseline and that were in focus in the images from subsequent time points for each eye were readily identified based on retinal features present in each of the images including blood vessels, fluorescent axon bundles, and relative positions of the few cells that retained their fluorescence throughout the study. In the eyes of mice that received vehicle cream cheese, mean fluorescent retinal cell counts were 36.4±4.9, 25.0±2.1, 19.8±4.0, and 17.3±3.3 cells per defined area at 1, 2, 3 and 4 weeks after optic nerve crush, respectively. In contrast, mean fluorescent retinal cell counts in the eyes of mice that received OT-440 in cream cheese were 39.2±4.0, 31.8±2.1, 30.2±1.1 and 27.7±2.2 cells per defined area at 1, 2, 3 and 4 weeks after optic nerve crush, respectively (Figure 3). When the counts at each time point were directly compared, the numbers of fluorescent cells at each time point were greater in the animals that received OT-440 by 7.5%, 27.3%, 52.3% and 60.1% than in corresponding control animals at 1, 2, 3 and 4 weeks after optic nerve crush, respectively (*P*<0.001 at weeks 2, 3 and 4).

The rate of decline in the counts of fluorescent cells was greater in the first two weeks after nerve crush than in the latter two weeks (Figure 3). Slope analysis of the results for the first two weeks revealed that OT-440 treatment significantly slowed the rate of fluorescent cell count reduction (*P*<0.001 at 2 weeks). After 2 weeks, the course of fluorescent cell count reductions in both groups were roughly parallel. Linear curve fitting analysis of the slopes defined by the data at weeks 2, 3 and 4 yielded r^2^ = 0.985 for the control results and r^2^ = 0.963 for the OT-440 results, indicating that slope analysis during this period was appropriate. The slope of the control data was −3.825±0.882 percent of baseline/week (standard error) and the slope of the OT-440 data was −2.08±0.546 percent of baseline/week. Analysis of covariance indicated these slopes were not significantly different.

### Quantitative Analysis: OT-440 Dissolved in Water

**Figure 4:**
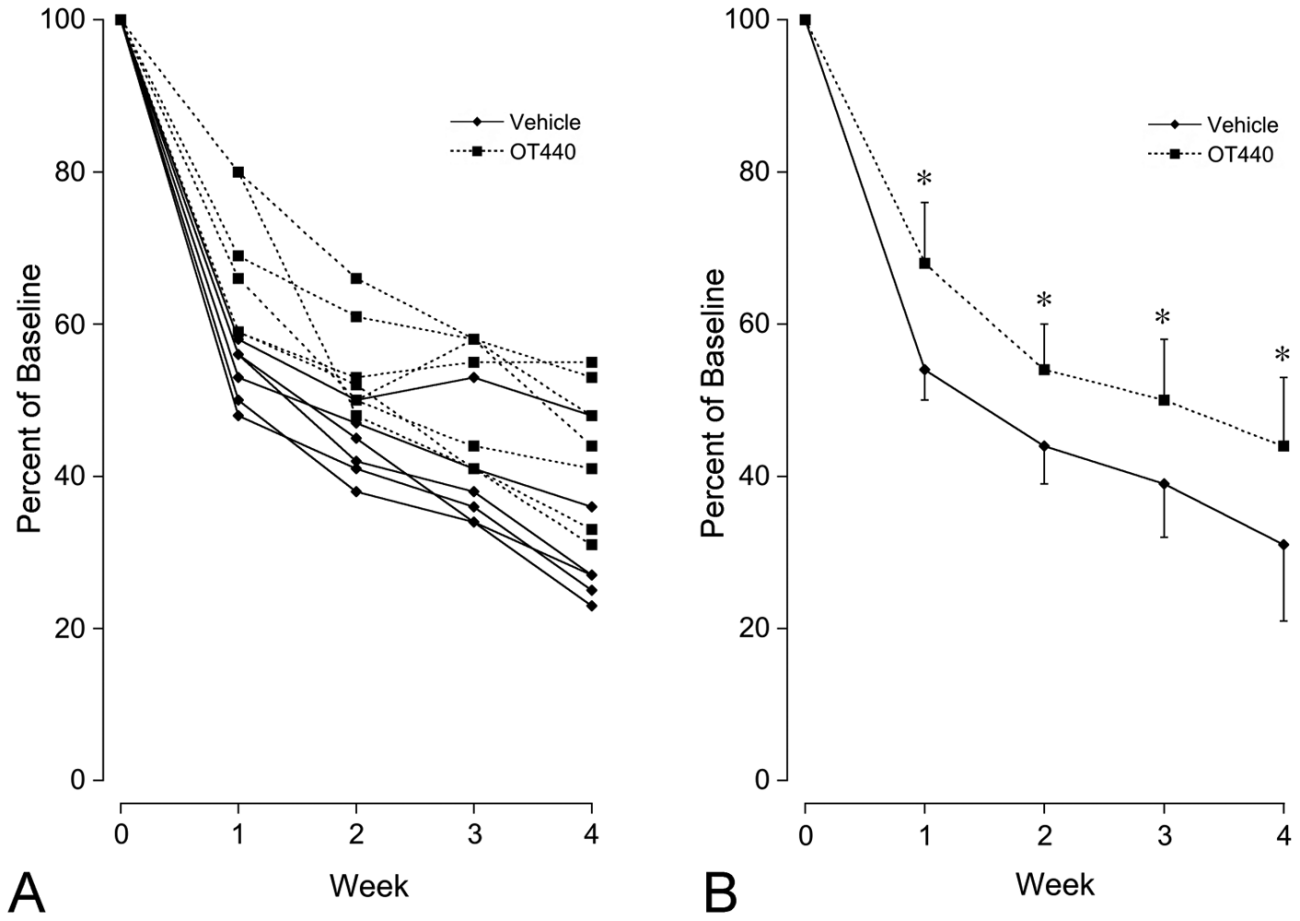
Loss of fluorescent neurons during the four weeks in defined retinal areas following optic nerve crush in mice that received water vehicle or OT-440 in water. Total data graph (**A**) shows that the general pattern for each was similar to the pattern in the cream cheese study. Mean data graph (**B**) shows significant differences between the vehicle and OT440 groups at weeks 1, 2, 3 and 4. N = 6 for all vehicle group data points and N = 7 for all OT-440 group data points. Asterisks indicate *P*<0.05 (students t-test). Error bars indicate SD.

In the eyes of mice that received water only, mean fluorescent retinal cell counts were 34.3±4.0, 28.0±2.9, 25.2±4.6, 19.8±6.2 cells per defined area at 1, 2, 3 and 4 weeks after optic nerve crush, respectively. In contrast, mean fluorescent retinal cell counts in the eyes of mice that received OT-440 in water were 43.7±5.4, 34.7±4.1, 32.3±5.3 and 27.9±5.9 cells per defined area at 1, 2, 3 and 4 weeks after optic nerve crush, respectively (Figure 4). When the counts at each time point were directly compared, the numbers of fluorescent cells at each time point were greater in the animals that received OT-440 by 27.3%, 23.9%, 28.3% and 40.5% than in corresponding control animals at 1, 2, 3 and 4 weeks after optic nerve crush, respectively (*P*<0.04 at all time points).

The pattern of cell count decline in the gavage results was similar to the pattern observed in the cream cheese study with faster loss during the first two week period after nerve crush and slower loss during the second two week period (Figure 4). The rate of fluorescent retinal neuron loss during the first two week period was significantly slower in the OT-440 group than in the vehicle group (*P*<0.001). In contrast, fluorescent retinal neuron loss was essentially parallel in the water only and OT-440 groups. Assessment of the data at weeks 2, 3 and 4 by slope analysis yielded r^2^ = 0.972 for the control results and r^2^ = 0.969 for the OT-440 results. The slope of the vehicle control data was −4.08±1.33 percent of baseline/week (standard error) and the slope of the OT-440 data was −4.14±1.39 percent of baseline/week. Analysis of covariance indicated these slopes were not significantly different.

### Body Weight

The high caloric content of cream cheese used in the first study had the potential to induce weight gain. Conversely, stress associated with gavage administration used in the second study might trigger weight loss. Because body weight changes might influence metabolism thereby affect the experimental results, the body weight of each animal was determined on a weekly basis. As shown in Table 2, body weight did not significantly change during the course of the study for any of the treatment groups (*P*>0.5 for each group, ANOVA).

**Table 2:** Change in body weight over the course of the study.

Experimental Group	Baseline*	Week 1	Week 2	Week 3	Week 4	Week 5	Week 6	ANOVA
CC Vehicle	23.3±2.9	23.6±2.5	24.1±2.5	24.1±2.0	24.5±1.9	24.6±1.9	23.9±2.1	*P* = 0.74
CC OT-440	23.9±3.1	24.3±2.4	25.3±2.5	25.3±2.5	25.6±2.5	26.0±2.5	25.4±2.4	*P* = 0.57
Water Vehicle	23.9±4.1	23.6±3.1	23.9±3.4	23.6±4.1	23.7±4.1	24.7±3.5	23.6±3.8	*P* = 0.99
Water OT-440	23.8±2.8	21.9±2.1	22.1±2.2	22.4±2.7	22.5±2.5	23.0±2.8	21.8±2.9	*P* = 0.64

Vehicles were cream cheese (CC) or water. Data expressed as mean grams body weight ± SD.

* Drug or vehicle administration started at the beginning of the first week and optic nerve crush occurred at the end of the second week. Hence, the end of study weeks 3, 4, 5 and 6 corresponded to 1, 2, 3 and 4 weeks after crush.

## Discussion

These results demonstrate that the number of fluorescent retinal cells in Thy1-CFP23Jrs mice is markedly greater in the animals that received OT-440 than in controls at 1, 2, 3 and 4 weeks following optic nerve crush. Direct comparison of the results at each time point showed there was up to 60% more fluorescent retinal neurons in the animals that received OT-440 than in corresponding control animals. The rate of decline in fluorescent cell counts was much greater in the first two weeks following optic nerve crush than after the second week. In both the cream cheese study and the water gavage study, many mice had robust protection detectable after the first week while other mice the robust protection was delayed by one week (panel A in Figures 3 and 4). As the temporal resolution of these studies was one week, it seems most appropriate to state that the major effect occurred during the first two weeks. During the second two week period, however, the benefit that had been attained during the first two weeks was maintained. Thus OT-440 appeared to have provided important protective benefit throughout the 4 week study period.

The absence of any change in the number of fluorescent cell counts in normal mice during two weeks of OT-440 treatment suggests that there was no direct effect on CFP expression. Likewise, the absence of significant change in body weight during the course of the study indicates that both the vehicle and the OT-440 treatments had minimal impact on the general metabolism of the study mice. Also, since the method used for optic nerve crush has been shown to preserve the retinal blood supply [14], the responses largely reflected RGC axon injury. Together, these results suggest the reduced rate of fluorescent cell count decline in the mice that received OT-440 primarily reflects a protective effect on CFP expression within initially fluorescent retinal neurons.

Similar increases in fluorescent retinal cell counts were observed in the mice that received OT-440 mixed with cream cheese and those that received aqueous OT-440 delivered by gavage. Greater variability in the results of the group that received OT-440 may reflect differences in pharmacokinetics associated with the different vehicles or dosing schedules. Alternatively, it could reflect normal experimental variability. Nevertheless, the increase in retinal fluorescent cells as a result of OT-440 treatment exceeded 40% for each treatment regimen. Thus, significant protection of CFP expression by oral OT-440 was obtained whether or not it was administered in the presence of food.

Retrograde tracer studies have shown that the brightly fluorescent fluorescent retinal neurons in normal Thy1-CFP23Jrs eyes were >97% RGCs [13]. However, immunohistochemical studies showed that 20% of CFP-expressing cells in healthy Thy1-CFP23Jrs mice are cholinergic amacrine cells [17]. These amacrine cells have substantially weaker fluorescence than the bright fluorescent retinal neurons in this mouse strain [17]. Also, they appear to be refractory to cell loss within a mouse line in which loss of RGCs is associated with gradual elevation of intraocular pressure [18]. Because the imager gain used during collection of the images after the crush was increased, it is likely that the fluorescent retinal neurons in these images included the CFP-expressing amacrine cells. In view of these considerations, it appears appropriate to estimate the loss of CFP-expressing RGCs in the current study by subtracting 20% of the baseline counts from the current observations. Performing this calculation estimates that fluorescent RGCs in the vehicle-treated mice gradually declined to ∼10% of baseline total fluorescent retinal neurons at the end of four weeks after crush (Table 3). This result is consistent with prior direct assessment of Thy-1 expression in RGCs after optic nerve crush [5]. In contrast, the estimated preservation of fluorescent RGCs in mice that received OT-440 was 2 -fold to 3-fold greater at 4 weeks after optic nerve crush. These estimates support the view that OT-440 can provide substantial protection to RGCs expressing Thy-1 following optic nerve crush.

**Table 3:** Estimated preservation of fluorescent RGCs*.

Group	Week 1	Week 2	Week 3	Week 4
CC Vehicle	37±8	19±3	11±6	7±5
CC OT-440	41±6	30±3	27±2	23±4
Water Vehicle	34±4	24±5	19±7	11±10
Water OT-440	48±8	34±6	30±8	24±9

* %± SD relative to total baseline fluorescent retinal neurons. Determined by subtracting 20% from the percent preservation of total fluorescent retinal neurons relative to baseline. CC, cream cheese.

Several studies have identified anti-oxidant properties for tempol and related tempol-based compounds. These compounds readily react with superoxide anion to form hydrogen peroxide in a manner similar to superoxide dismutase [19], [20]. In brain, tempol has been shown to also catalytically scavenge peroxynitrite-derived nitrogen dioxide radicals and carbonate radicals [21]. As mentioned above, the tempol analogue OT-440 inhibits lipid peroxidation within human THP-1 monocytic leukemia cells exposed to lipopolysaccharide at concentrations similar to the expected tissue concentrations of the present study [22]. Unknown is whether altered Muller cell or microglial activation might contribute to the observed protective effect [23]–[27]. Nonetheless, these observations collectively support the view that increased number of fluorescent RGCs following optic nerve crush in mice that received treatment with OT-440 involves protection from early oxidative RGC changes that would otherwise occur following optic nerve injury.

In conclusion, the results of the present study demonstrate that OT-440 treatment protects against loss of CFP expression that occurs with early RGC damage following optic nerve injury in Thy1-CFP23Jrs mice. These results justify further studies to clarify the mechanism of this effect in RGCs, as well as to determine if OT-440 can protect against optic nerve damage or RGC loss due to intraocular pressure elevation.

## Materials and Methods

### Ethics Statement

All experimental procedures conformed to the ARVO Statement for the Use of Animals in Ophthalmic and Vision Research. The protocol was approved by the Institutional Animal Use and Care Committee of the University of California San Diego (Protocol Number: S98096).

### Animals

This study used hemizygous B6.Cg-Tg (Thy1-CFP)23Jrs mice (both male and female) approximately 6–7 months old. These animals were bred at UCSD from the same stocks that provided animals for prior studies [13], [14].

### Treatments

In parallel experiments, OT-440 treatments were delivered as a supplement in a food that mice will readily eat to mimic a conventional oral drug, or as a gavaged aqueous preparation to more closely regulate dosage. OT-440 was formulated with cream cheese for feeding treatments or with water for gavage treatments by Othera Pharmaceuticals, Inc. Pharmacokinetic studies showed that 0.006% of a single oral OT-440 dose distributes to the brain by 30 minutes after administration (G. Patil, unpublished, 2010). Because adult mouse brain contains 80% water [28], [29] and adult C57BL/6 mouse (the background strain) brains weigh 0.41–0.45 grams [30], [31], this result suggests the concentration of OT-440 in the water of the brain following a 5 mg oral dose is 4 µM. This dose also resulted in OT-440 concentrations of 11 µM in retina/choroid and 20 µM in optic nerve tissue. Incubation of cultured THP-1 cells with 1 µM or 10 µM OT-440 (compound 4) reduced lipopolysaccharide-induced lipid peroxidation by 87% and 91%, respectively [22]. Hence, to obtain OT-440 concentrations in the retina and optic nerve similar to this in vitro study, the total daily treatment dosage for this study was chosen to be 5 mg/dose.

To make the experimental cream cheese treatment, OT-440 was dissolved in 6–7 mL of water and mixed with 50 g Philadelphia brand cream cheese (Kraft Foods, Northfield, IL) to achieve a final dose of 1.5% (w/w). Vehicle control was plain cream cheese. For presentation, 0.1 g of experimental or control cream cheese was placed into a low-density polyethylene plastic container (Part No. 0801–07, SKS Bottle & Packaging, Watervliet, NY) labeled by a letter code and stored refrigerated (4°C) until used. HPLC studies by Othera, Inc. did not detect any degradation of OT-440 for at least 5 days after mixing with cream cheese (data not shown). These treatments and corresponding vehicle treatments were prepared, labeled by code, and then sent with cool packs to the University of California San Diego by overnight courier. For dosing, the person presenting the cream cheese cups was masked to whether the cream cheese doses were experimental or control. Cream cheese containers for each feeding were brought to room temperature prior to presentation. For treatment, each animal was transferred to a cage containing one container of cream cheese fastened onto the inside surface of the cage about 2 cm above the floor. The mice typically consumed the cream cheese within 5 minutes after presentation. Complete consumption of the cream cheese was confirmed prior to returning the mouse to its home cage. Doses of 1.66 mg each were provided three times daily at 7∶30 am, 1∶30 pm, and 7∶30 pm to yield a total daily treatment of 5 mg/day.

The vehicle for the gavage treatments was USP water for irrigation, adjusted with 0.1 N HCl to pH 3.0 and filtered through a 0.2-micron filter. Experimental gavage fluid contained 1.5% (w/v) OT-440 dissolved in vehicle and yielded a clear fluid. This treatment fluid and plain vehicle treatments were prepared by Othera Pharmaceuticals, placed in sealed containers that were labeled by code, and then sent with cool packs to the University of California San Diego by overnight courier. Because of the coding, the person administering the gavage treatments was masked to whether each dose contained the drug or vehicle only. Gavage doses of 2.5 mg in 0.2-mL were provided twice daily at 10∶30 am and at 7∶30 pm to yield a total daily treatment of 5 mg/day.

### bCSLO Imaging

Imaging by bCSLO was performed as previously described [13], [14]. Animals were gently restrained by hand and no anesthesia was used. A single scan corresponding to a retinal area of about 2 mm^2^ was obtained in less than 5 seconds. The short exposure period needed for focusing and image collection was generally well tolerated by the mice. Both eyes were imaged at each imaging session. This allowed monitoring of any fluorescent cell loss for reasons unrelated to the optic nerve crush. During pilot studies, we noticed that image counts occasionally would be reduced from week to week due to areas of poor cell resolution and that this corresponded with the appearance of small corneal scratches. These scratches often healed within a week. We also found that if the instrument gain setting was optimized at each imaging session, we obtained more consistent counts from week to week from the images of untreated animals. Hence, gain optimization was performed each time an animal was imaged. For each imaging session, sets of 5 to 8 images were obtained from different areas of the retina. These were quickly evaluated and if one or more of these images were out of focus or had some other technical problem (e.g. eye movement during image capture), repeat images were obtained.

### Experimental Design

Ocular biomicroscopic examination was performed to exclude any animals with abnormal ocular appearance. Power analysis based on data from our prior optic nerve crush study indicated 80% power to detect a 30% difference in the presence of a standard deviation of 8% with *P*<0.05 would be achieved if the sample size was 5/group or greater [14]. In anticipation of some losses due to technical issues, animals with normal ocular appearance were randomly assigned to each treatment group until 10 animals/group was attained. Average body weight for all animals was 23.6±3.1 grams and the means for each of the groups differed from this value by less than 3%. Next, retinal imaging by bCSLO was performed and then treatments with OT-440 or vehicle were initiated for all groups. Two weeks later, bCSLO images were collected to determine if there was any direct drug effect on CFP expression. Then monocular intraorbital optic nerve crush was performed as previously described [13], [14]. The crush duration was 5 seconds and care was taken to perform the crush sufficiently distal to the globe so that retinal circulation was preserved. We have previously confirmed by fluorescence angiography that our method preserves retinal circulation [14]. A sham crush procedure, during which the surgical field was exposed and then closed, was performed on the contralateral eye. Treatments continued uninterrupted and the animals were imaged by bCSLO at 1, 2, 3 and 4 weeks after crush. Body weight of each included animal was measured at the time of baseline image collection and then measured weekly for the remainder of the study. After initiation of the study, 3–4 enrolled animals/group were excluded due to the development of cataract or corneal opacity during the course of the study, or due to other technical difficulties. Hence, each group contained 6–7 animals that completed the study.

### Analysis

All of the images from a particular eye were reviewed at the end of the experiment to identify retinal regions in which there is good quality imaging of the fluorescent cells from each of the examination sessions. Selected region images meeting this criteria and containing 64 fluorescent cells at baseline were identified for each eye based on retinal features including blood vessels, fluorescent axon bundles, and the relative positions of the few cells that retained their fluorescence throughout the study. Using Photoshop (CS4, Adobe, San Jose, CA), these defined areas were cut from each image and the new file names were coded to mask their origin. Masked counts of fluorescent cells in these defined retinal areas were made by a counter unaware of the experimental condition or time point of the images. After unmasking, the counts at each time point for each eye were expressed as percent of the corresponding baseline count.

### Statistical Evaluation

Body weight and fluorescent cell count results were statistically analyzed using analysis of variance (ANOVA) to identify differences between parallel data sets, the students t test to directly compare differences between groups at each time point, and the paired students t test to compare differences between data pairs within two groups. Slopes of cell count reductions between 2 and 4 weeks after optic nerve crush were determined using Cricket Graph 1.3.2 (Cricket Software, Malvern, PA). Analysis of covariance was conducted using online software by H. Arsham (University of Baltimore, Baltimore, MD) [32].
